# Highly Efficient Removal of Barium(II) from Nuclear Wastewater by Calcined Magnesium–Aluminum Layered Double Hydroxides

**DOI:** 10.3390/toxics14050432

**Published:** 2026-05-14

**Authors:** Jun Wang, Zhaoxu Sun, Ziyi Liu, Xinglei Li, Yi Zhou, Ningchao Zheng, Qiang Wu, Chen Xu, Lu Gao, Hiroshi Watabe, Yanliang Chen, Yuezhou Wei, Xiangbiao Yin

**Affiliations:** 1 School of Nuclear Science and Technology, University of South China, 28 Changsheng West Road, Hengyang 421001, Chinazhengnch3@usc.edu.cn (N.Z.);; 2 School of Economics, Management and Law, University of South China, 28 Changsheng West Road, Hengyang 421001, China; 3 Research Center for Accelerator and Radioisotope Science (RARiS), Tohoku University, 6-3 Aoba, Aramaki, Aoba-ku, Sendai 980-8578, Japan; watabe@cyric.tohoku.ac.jp; 4 Shanghai Institute of Measurement and Testing Technology Co., Ltd., Shanghai 200233, China; 5 School of Nuclear Science and Engineering, Shanghai Jiao Tong University, 800 Dong Chuan Road, Shanghai 200240, China

**Keywords:** barium immobilization, layered double hydroxides, calcination, mediated mineralization, radioactive wastewater

## Abstract

Radioactive Ba^2+^ poses significant risks to nuclear safety and environmental protection, yet its efficient removal from nuclear wastewater remains a considerable challenge. Herein, Mg-Al layered double hydroxides (LDHs) were synthesized via a co-precipitation method and systematically optimized by tuning the Mg/Al molar ratio and calcination temperature. The optimal material, obtained by calcining Mg-Al LDH with a Mg/Al ratio of 4:1 at 450 °C (denoted as HT-450), exhibited a high apparent Ba^2+^ uptake capacity of 416 mg g^−1^ and reached equilibrium within 15 min. Structural and spectroscopic analyses indicate that Ba^2+^ immobilization is more appropriately described as a reconstruction-coupled, interfacially mediated mineralization process, in which insoluble BaCO_3_ forms in close association with the reconstructed HT-450 surface rather than through simple reversible adsorption or ion exchange. HT-450 also exhibited stable performance over a wide pH range of 3–7, high selectivity toward Ba^2+^ in the presence of competing mono-, di-, and trivalent cations, and excellent radiation tolerance, retaining approximately 95% of its initial uptake capacity after exposure to 200 kGy high-energy electron irradiation. These results demonstrate that HT-450 is a promising candidate for the rapid and stable immobilization of Ba^2+^ from Ba-containing radioactive wastewater.

## 1. Introduction

Against the backdrop of the increasing depletion of conventional fossil fuels, nuclear energy—characterized by high energy density and cost-effectiveness—has developed rapidly and now plays an important role in electricity generation [1,2]. However, the expansion of nuclear power plants has also intensified the challenge of managing radioactive wastewater generated during the nuclear fuel cycle. Among the various radionuclides of concern, ^133^Ba is particularly hazardous due to its relatively long half-life (10.5 years) and the emission of energetic γ-rays (356 keV). More critically, its chemical similarity to calcium promotes its accumulation in bone tissue and subsequent irradiation of bone marrow, thereby increasing the risks of leukemia and bone cancer [3,4,5]. Therefore, the effective removal and stable immobilization of radioactive Ba^2+^ from nuclear wastewater are of great significance for nuclear safety and environmental protection [5,6].

Various techniques have been employed for the removal of Ba^2+^ from aqueous systems, including ion exchange, membrane filtration, coagulation, precipitation, and adsorption [7,8,9,10,11]. Compared with other methods, adsorption offers several advantages for the removal of Ba^2+^ ions from wastewater, such as high efficiency, strong treatment capacity, and low cost [7,12,13]. A wide range of adsorbents, including bio-nanocomposites, activated carbon, dolomite, clays, hydrous ferric oxides, titanate nanofibers, and modified zeolites, have been investigated [6,14,15,16,17,18]. Nevertheless, many conventional adsorbents rely predominantly on reversible electrostatic interactions or ion-exchange mechanisms, which may increase the risk of remobilization, structural degradation under irradiation, and limited long-term stability [19,20]. For radioactive wastewater treatment, such reversibility may compromise long-term retention and environmental safety; therefore, the development of immobilization pathways capable of converting aqueous Ba^2+^ into stable solid phases is highly desirable [21].

In recent years, layered double hydroxides (LDHs) have attracted extensive attention as adsorbents for the removal of metal cations from aqueous solutions because of their low cost, simple synthesis, and excellent performance [22,23]. LDHs are a class of layered hydroxide materials with unique physicochemical properties arising from their well-defined lamellar structure, including tunable host metal cation layers, exchangeable interlayer anions, a pronounced structural “memory effect,” and micro/mesoporous characteristics [24,25]. Their general chemical formula can be represented as:

[M^2+^_1*−x*_M^3+^*_x_*(OH)_2_]^x+^(A^n−^)*_x_*/*_n_*·*m*H_2_O,

where M^2+^ and M^3+^ denote divalent and trivalent metal cations in the brucite-like host layers, respectively, and A^n−^ represents charge-balancing interlayer anions [26]. The parameter *x* is the molar fraction of M^3+^ cations and determines the M^2+^/M^3+^ ratio, which is commonly in the range of 2–4, while *m* is the number of moles of interlayer water molecules [27]. Despite these advantages, pristine LDHs still suffer from limited uptake capacity and insufficient structural stability, which restrict their practical application in wastewater treatment.

To overcome these drawbacks and further enhance adsorption performance, a variety of modification strategies has been developed, including high-temperature calcination, intercalation modification, and the formation of LDH-based composites [28,29]. Such modifications can effectively tune the surface properties, pore structure, and chemical composition of LDHs, thereby improving their affinity toward target pollutants [30]. In particular, when LDHs are calcined at elevated temperatures, they are transformed into layered double oxides (LDOs) or mixed metal oxides with defect-rich and highly reactive surfaces [31]. Upon rehydration in aqueous media, these calcined products can partially reconstruct into LDH-like structures through the memory effect, while simultaneously generating newly accessible interfacial sites that facilitate rapid ion capture and subsequent immobilization [32,33,34]. As a result, calcination–rehydration treatment often leads to markedly improved removal performance. Previous studies have also shown that calcination at appropriate temperatures can enhance the uptake capacity of LDH-based materials for various metal ions [27]. In addition, the M^2+^/M^3+^ molar ratio plays a crucial role in determining the adsorption properties. In general, increasing the M^2+^/M^3+^ ratio decreases the layer charge density, which may expand the interlayer spacing and thereby influence the adsorption performance. The nature of the interlayer anions also significantly influences the LDH structure and, in turn, the removal efficiency toward specific target ions [35,36].

However, most existing LDH-based studies have focused on the removal of transition metal or actinide ions, and the reported mechanisms are predominantly attributed to surface adsorption or ion exchange [37,38]. In contrast, the application of calcined LDHs for the stable immobilization of alkaline-earth metal ions such as Ba^2+^ has received comparatively little attention. Unlike many transition metal ions, Ba^2+^ exhibits relatively weak complexation with common surface functional groups but readily reacts with carbonate species to form thermodynamically stable and sparingly soluble BaCO_3_. Consequently, conventional adsorption or ion-exchange strategies may be insufficient to ensure stable Ba^2+^ retention, particularly under dynamic or irradiated conditions. From this perspective, developing immobilization pathways that convert aqueous Ba^2+^ into stable solid phases through interfacially mediated mineralization processes is especially attractive. Unlike conventional homogeneous precipitation induced by soluble carbonate salts such as Na_2_CO_3_ or K_2_CO_3_, HT-450 acts as a reconstructable solid matrix that couples BaCO_3_ formation with interfacial immobilization. This feature is advantageous because it integrates capture, solid-phase stabilization, and subsequent solid–liquid separation within one material system, thereby potentially reducing the risk of forming freely suspended precipitates and secondary remobilization.

**Figure 1:**
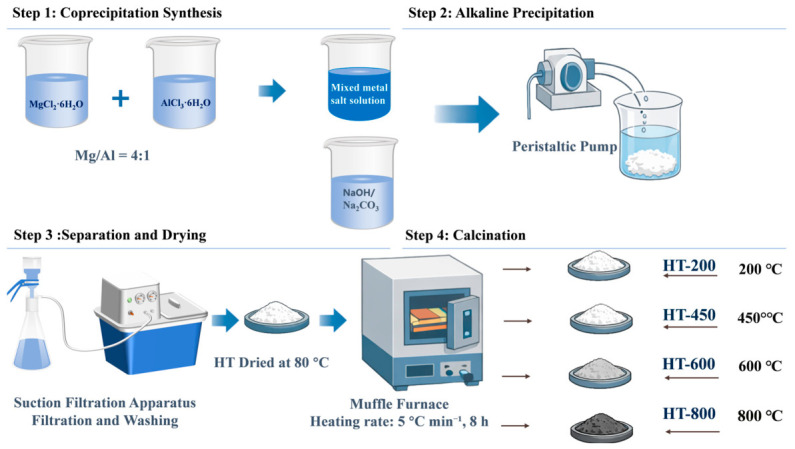
Schematic illustration of the procedure for synthesizing HT.

Herein, we report a rationally designed Mg-Al LDH material (denoted as HT-t) for efficient and radiation-resistant Ba^2+^ immobilization. The synthesis procedure for HT-t is shown in Figure 1. By optimizing composition and calcination conditions, HT-450 achieves rapid and selective Ba^2+^ capture. Mechanistic analyses indicate that Ba^2+^ immobilization is more appropriately described as a reconstruction-coupled, interfacially mediated mineralization process, in which BaCO_3_ forms in close association with the reconstructed HT-450 surface and contributes to stable immobilization. The excellent radiation tolerance of the material further highlights the potential of this strategy for the treatment of Ba-containing radioactive wastewater.

## 2. Experimental Section

### 2.1. Chemicals

Anhydrous ethanol (EtOH, AR), sodium hydroxide (NaOH, AR), sodium carbonate (Na_2_CO_3_, 99.9%), magnesium chloride hexahydrate (MgCl_2_·6H_2_O, 98%), and sodium chloride (NaCl, 99.5%) were purchased from Sinopharm Chemical Reagent Co., Ltd. (Shanghai, China). Aluminum chloride hexahydrate (AlCl_3_·6H_2_O, 97%), anhydrous calcium chloride (CaCl_2_, 96%), iron chloride hexahydrate (FeCl_3_·6H_2_O), and barium chloride (BaCl_2_, 99%) were obtained from Shanghai Macklin Biochemical Co., Ltd. (Shanghai, China). Deionized water with a resistivity of 18.25 MΩ·cm was used throughout this study for solution preparation and all tests.

### 2.2. Synthesis Procedure of HT

Mg-Al layered double hydroxide (LDH) with a Mg/Al molar ratio of 4:1 was synthesized by a coprecipitation method. At room temperature, MgCl_2_·6H_2_O (18.67 g) and AlCl_3_·6H_2_O (7.47 g) were each dissolved in 100 mL of deionized water and magnetically stirred at 1000 rpm for 5 min. The two solutions were then mixed and stirred continuously to form a homogeneous mixture. Meanwhile, an alkaline solution was prepared by dissolving NaOH (8 g) and Na_2_CO_3_ (0.53 g) in deionized water. The mixed metal salt solution was added dropwise to the alkaline solution using a peristaltic pump at a constant flow rate of 5 mL min^−1^. The precipitate was filtered, washed several times with a 0.2 M Na_2_CO_3_ solution, dried in an oven at 80 °C for 20 h, and finally sieved to obtain a homogeneous solid powder.

The synthesized Mg-Al LDH powder was then placed in the center of a muffle furnace. The furnace was heated to different target temperatures (200, 450, 600, and 800 °C) at a constant heating rate of 5 °C min^−1^ and maintained at each target temperature for 8 h. The resulting calcined samples are designated as HT-t, where t denotes the specific calcination temperature. The uncalcined, as-synthesized Mg-Al LDH is denoted as HT.

### 2.3. Characterization

The surface morphology of the samples was characterized by scanning electron microscopy (SEM) using a Thermo Fisher Scientific Pharos G2 instrument (Waltham, MA, USA) equipped with an energy-dispersive X-ray spectroscopy (EDS) detector. For transmission electron microscopy (TEM) analysis, samples were dispersed in ethanol via ultrasonication and deposited onto holey-carbon-coated copper grids to examine their interlayer structure and micro-morphology. Fourier-transform infrared (FT-IR) spectra were recorded on a Shimadzu IR Tracer-100 spectrometer (Tokyo, Japan) in the range of 4000–400 cm^−1^ with a resolution of 4.0 cm^−1^. X-ray diffraction (XRD) patterns were obtained using a Rigaku Smart Lab-SE diffractometer with Cu Kα radiation (λ = 1.5406 Å), scanning from 10° to 80° at a rate of 10° min^−1^. Crystallinity was calculated with the MDI Jade 6 software. Specific surface area and porosity were determined by N_2_ physisorption at 77 K on a Micromeritics ASAP 2460 analyzer(Micromeritics Instrument Corp., Norcross, GA, USA). Prior to measurement, samples were degassed under vacuum at 80 °C for 15 h. The Brunauer–Emmett–Teller (BET) method and the Barrett–Joyner–Halenda (BJH) model were applied to derive the specific surface area and pore-size distribution, respectively. Chemical composition and surface electronic states were analyzed by X-ray photoelectron spectroscopy (XPS) on a Thermo Scientific K-Alpha system with a monochromatic Al Kα source (1486.6 eV).

### 2.4. Adsorption and Desorption Experiments

Adsorption experiments: For adsorption kinetics studies, 20 mg of HT-t was added into a 50 mL centrifuge tube containing 20 mL of Ba^2+^ solution (1000 mg/L). The tube was then agitated on a constant-temperature shaker at 25 °C and 160 rpm. Except for kinetic experiments, all adsorption tests were conducted for a duration sufficiently to reach equilibrium. After adsorption, the HT-t was separated from the solution by filtration through a 0.45 μm syringe filter. The equilibrium concentration of Ba^2+^ in the filtrate was measured by inductively coupled plasma optical emission spectrometry (ICP-OES) (Ultima-E, HORIBA, Kyoto, Japan). The adsorption capacity (Q) and removal percentage (R) of Ba^2+^ were calculated using Equations (1) and (2).

(1) $$Q=\frac{\left(C_{0}-C_{e}\right)\times V}{M}$$

(2) $$R=\frac{C_{0}-C_{e}}{C_{0}}\times 100\%$$

where Q (mg g^−1^) is the adsorption capacity of HT-t for Ba^2+^; C_0_ and C_e_ (mg/L) are the initial and equilibrium concentrations of Ba^2+^, respectively; M (g) is the mass of HT-t used, and V (L) is the volume of the Ba^2+^ solution.

Desorption experiments: After adsorption saturation, 0.02 g of the spent adsorbent was immersed in 20 mL of a desorbing solution. The Ba^2+^ desorption performance of different desorbing agents was evaluated. Additionally, 0.02 g of the spent adsorbent was uniformly dispersed in 20 mL of deionized water and shaken at 25 °C for seven days. The concentration of Ba2+ released into the aqueous phase was measured to examine whether the adsorption on HT-t was irreversible. The desorption efficiency (D) was calculated using Equation (3).

D = (C_e_ × V)/(m × Q) × 100% (3)

where m (g) is the mass of spent adsorbent, V (L) is the volume of the desorbing solution, C_e_ (mg/L) denotes the equilibrium concentration of Ba^2+^ in the desorption solution, and Q (mg g^−1^) represents the equilibrium adsorption capacity.

## 3. Results and Discussion

### 3.1. Characterization of HT-t

**Figure 2:**
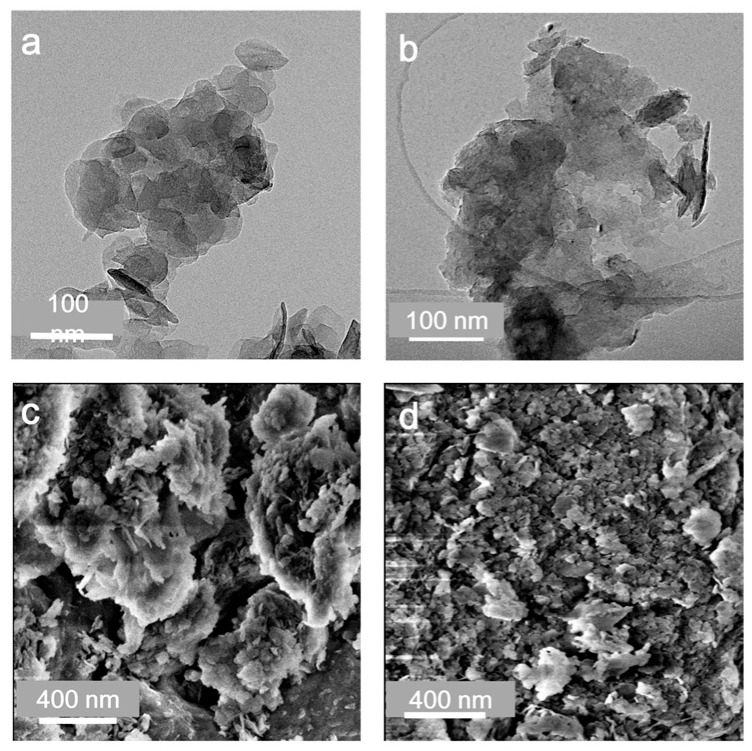
TEM images of (**a**) HT and (**b**) HT-450. SEM images of (**c**) HT and (**d**) HT-450.

TEM and SEM observations collectively reveal the morphological evolution of HT upon calcination. As shown in Figure 2a,c, the pristine HT consists of aggregated plate-like particles with partial lamellar features, although the layered structure cannot be completely resolved with full clarity, the overall morphology remains broadly consistent with that reported for Mg-Al hydrotalcite materials in the literature [39]. After calcination at 450 °C, the original lamellar features become less distinguishable: the nanosheets appear thicker and less transparent, accompanied by curling, collapse, and aggregation (Figure 2b). Consistently, Figure 2d shows that HT-450 exhibits a rougher and more compact surface composed of fragmented and aggregated oxide particles, indicating partial collapse of the parent LDH framework and the formation of mixed Mg-Al oxides.

**Figure 3:**
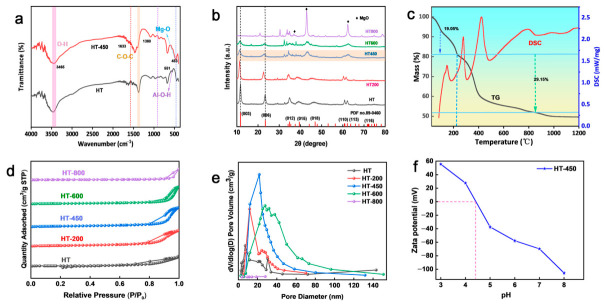
(**a**) FT-IR spectra of HT and HT-450. (**b**) XRD patterns of the five HT samples. (**c**) TG-DSC curves of HT-450. (**d**) N_2_ adsorption–desorption isotherms of the five HT samples. (**e**) Pore diameter distributions of the five HT samples. (**f**) Zeta potential of HT-450 at different pH values.

Figure 3a shows that both HT and HT-200 display sharp, well-defined hydrotalcite-type reflections, indicating that the layered framework remains largely intact below 200 °C. This observation corresponds to the first mass-loss step (~19% below ~220 °C) in the TG curve (Figure 3c), which is accompanied by an endothermic DSC signal and is primarily ascribed to the removal of physisorbed and interlayer H_2_O without significant disruption of the lamellar order [40]. The FT-IR spectrum of pristine HT (Figure 3a) further supports this interpretation, showing a broad O-H stretching band at ~3465 cm^−1^ and an H-O-H bending vibration at ~1633 cm^−1^, together with a distinct ν_3_ carbonate band at ~1380 cm^−1^, confirming the presence of interlayer CO_3_^2−^ within the hydrotalcite framework [41,42].

As shown in Figure 3b, the XRD reflections of HT-450 become broadened and weakened, indicating partial collapse of the layered structure and decreased structural ordering due to dehydroxylation and incipient carbonate decomposition [43]. This aligns with the second major mass-loss step (~29%) between 220 °C and 600 °C in the TG curve, which corresponds to clear thermal events in the DSC profile [41,44]. The FT-IR spectrum of HT-450 confirms this structural change: the O-H stretching and H-O-H bending bands are substantially reduced, and the carbonate peak near 1380 cm^−1^ is markedly weakened, reflecting substantial removal of interlayer H_2_O and partial loss of interlayer CO_3_^2−^, together with disruption of the layered framework. Nevertheless, metal–oxygen lattice vibrations in the 600–400 cm^−1^ region remain observable, suggesting the formation of thermally derived mixed Mg-Al oxide phases while the basic metal–oxygen skeleton is retained [45]. At 800 °C, the characteristic LDH reflections nearly vanish, and new peaks corresponding to crystalline MgO emerge, confirming the transformation of the layered structure into mixed oxide phases [46,47]. These results suggest that HT-450 represents an intermediate activated state in which the original lamellar framework is largely disrupted, while a reactive mixed Mg-Al oxide structure capable of subsequent reconstruction is preserved.

The N_2_ adsorption–desorption isotherms and pore-size distributions of the five HT samples are presented in Figure 3d,e. Among them, HT-800 exhibits an essentially featureless isotherm with negligible uptake, indicating severe collapse of the porous structure at 800 °C and a much lower accessible surface area [48]. The BET surface areas of HT, HT-200, HT-450, HT-600, and HT-800 are 16.31, 44.21, 48.75, 28.26, and 12.64 m^2^ g^−1^, respectively, while BJH analysis yields corresponding average pore diameters of 9.4, 12.4, 16.3, 23.5, and 10.3 nm. Thus, the specific surface area initially rises with calcination temperature, reaching a maximum for HT-450, and then declines upon further heating, whereas the pore size gradually increases with increasing calcination temperature up to 600 °C, followed by a decrease at 800 °C. The superior textural properties of HT-450 suggest that calcination at 450 °C generates a more open and accessible mixed-oxide structure without causing excessive sintering, which is favorable for subsequent hydration-driven reconstruction and interfacial Ba^2+^ immobilization.

Figure 3f shows that the zeta potential decreases monotonically from positive values under acidic conditions to negative values at higher pH, with an isoelectric point (IEP) near pH 5.6. This result suggests that electrostatic attraction may facilitate the initial interfacial approach of Ba^2+^ under suitable pH conditions, but it is unlikely to be the dominant factor governing the final immobilization pathway.

### 3.2. Adsorption Behavior of Ba^2+^ by HT-t

A systematic optimization was performed to identify the most effective LDH-based material for Ba^2+^ removal. As shown in Figure S1, Mg-Al LDH exhibited markedly higher Ba^2+^ uptake than Ca-Al, Mg-Fe, and Ca-Fe LDHs, both before and after calcination, and was therefore selected as the parent system for further study. The Mg/Al ratio was then optimized (Figure S2). The uptake capacity increased from 315 mg g^−1^ at 2:1 to 352 mg g^−1^ at 3:1, reached a maximum of 416 mg g^−1^ at 4:1, and decreased to 298 mg g^−1^ at 6:1, demonstrating the strong dependence of Ba^2+^ removal on precursor composition. Accordingly, a Mg/Al ratio of 4:1 was selected. The effect of precursor interlayer anions was also examined using calcined LDHs derived from CO_3_^2−^, SO_4_^2−^, and OH^−^-containing precursors (Figure S3). Given their distinct thermal stability and decomposition behavior, the precursor anions are expected to influence the structural evolution and reconstruction characteristics of the calcined products.

**Figure 4:**
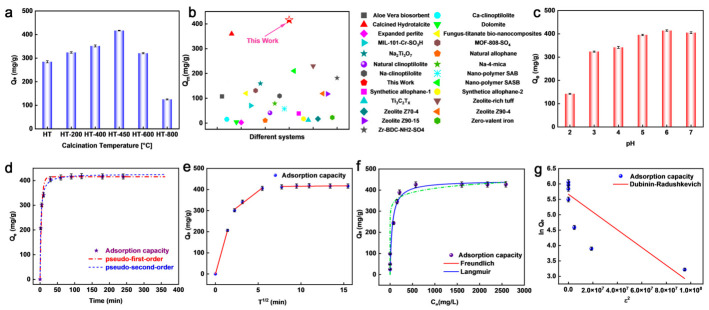
(**a**) Effect of temperature and (**b**) Comparison of the maximum Ba^2+^ adsorption capacities of HT-450 with previously reported adsorbents (detailed references can be seen in Table S1). (**c**) Effect of pH on the adsorption capacity of Ba^2+^ by HT-450. (**d**) Effect of contact time on Ba^2+^ adsorption, with kinetic behavior fitted by pseudo-first-order and pseudo-second-order models. (**e**) Intraparticle diffusion model fitting. (**f**,**g**) Adsorption isotherms of Ba^2+^ on HT-450 and corresponding fitting results using Langmuir, Freundlich, and Dubinin–Radushkevich models.

The adsorption performance of the optimized materials was further compared. As shown in Figure 4a, HT-450 exhibited the highest Ba^2+^ uptake among all HT samples, highlighting 450 °C as the optimal calcination temperature. This enhancement is attributed to the generation of a more reactive mixed Mg-Al oxide with favorable textural properties, whereas calcination at 800 °C caused severe structural collapse and particle agglomeration, resulting in a marked loss of performance. A similar trend was observed for the precursor-anion series, in which the carbonate-derived sample showed the highest uptake capacity (Figure S3). As shown in Figure 4b, HT-450 also exhibited a markedly higher Ba^2+^ uptake capacity than most previously reported adsorbents under comparable conditions. Compared with various natural minerals, modified clays, zeolites, and bio-based composites, HT-450 demonstrated superior Ba^2+^ removal performance, highlighting its strong competitiveness among currently available Ba^2+^ sorbents. These results confirm that both cation composition and precursor anion chemistry govern the final Ba^2+^ removal performance, and identify HT-450 derived from the carbonate-form precursor as the optimal adsorbent for subsequent study.

The effect of initial pH on Ba^2+^ uptake by HT-450 is shown in Figure 4c. The uptake capacity increased from pH 3 to 6 and reached a maximum of 416 mg g^−1^ at pH 6, reflecting reduced proton competition, improved structural stability, and more favorable conditions for interface-mediated immobilization [49,50]. At higher pH, the uptake capacity decreased, indicating that electrostatic interaction alone cannot account for the overall uptake behavior [51,52]. To evaluate possible homogeneous BaCO_3_ precipitation, blank experiments without HT-450 were conducted at different initial pH values under otherwise identical conditions. As shown in Figure S4, the Ba^2+^ concentration remained essentially unchanged from pH 2 to 7, with no visible or recoverable precipitate, indicating negligible homogeneous BaCO_3_ precipitation within the pH range used for adsorption and mechanistic analyses. Noticeable Ba^2+^ loss occurred only at pH 8, suggesting that homogeneous precipitation becomes relevant only under more alkaline conditions. In contrast, acidic conditions destabilized the HT framework, as evidenced by the release of 5.26 mg L^−1^ Mg and 4.27 mg L^−1^ Al at pH 4. These results indicate that slightly acidic to neutral conditions are more suitable for evaluating the intrinsic Ba^2+^ immobilization performance of HT-450, consistent with previous reports on HT-based materials for alkaline-earth metal uptake.

The uptake kinetics of Ba^2+^ on HT-450 were analyzed using pseudo-first-order, pseudo-second-order, and intraparticle diffusion models. As shown in Figure 4d, the uptake process proceeds rapidly in the initial stage, reaching more than 85% of the equilibrium capacity within the first 10 min and approaching equilibrium after approximately 15 min. The pseudo-second-order model provides a better fit than the pseudo-first-order model (Table S2), indicating that the early-stage uptake behavior is dominated by rapid surface-associated interactions. However, this kinetic model should be regarded only as an empirical description of the apparent uptake behavior and does not by itself distinguish among different pathways, including surface-associated adsorption, surface complexation, and precipitation-coupled immobilization [53].

The intraparticle diffusion plot further suggests a multi-stage uptake behavior (Figure 4e). The Weber–Morris model is an empirical approach for evaluating the possible contribution of intraparticle diffusion, and its segmented linear regions should not be overinterpreted as direct evidence for distinct microscopic steps [54,55]. The fitted lines do not pass through the origin, indicating that intraparticle diffusion may participate in the overall uptake process but is unlikely to be the sole rate-controlling step. Three approximately linear regions can be identified, which may be tentatively associated with rapid external surface-associated uptake, diffusion-assisted transport into less accessible pores or internal regions, and gradual approach to equilibrium. This assignment should therefore be regarded as a tentative kinetic interpretation, and further particle-size-dependent, stirring-rate-dependent, or time-resolved characterization studies would be needed to rigorously distinguish the individual transport and immobilization steps.

The adsorption isotherms of Ba^2+^ on HT-450 were analyzed using the Langmuir, Freundlich, and Dubinin–Radushkevich (D-R) models. As shown in Figure 4f, the uptake capacity increased rapidly at low equilibrium concentrations and gradually approached saturation with further increasing concentration. Among the tested models, the Langmuir equation provided the best fit (Table S3). However, this result is interpreted here as an empirical description of the apparent uptake behavior rather than direct evidence for ideal monolayer adsorption on a homogeneous surface. The calculated maximum uptake capacity (Q_m_) of 421.1 mg g^−1^ was in good agreement with the experimental value of 416 mg g^−1^, confirming the high apparent Ba^2+^ uptake capability of HT-450.

The D-R model gave a relatively low correlation coefficient (R_2_ = 0.77; Figure 4g), indicating its poor applicability to the present system. Therefore, the D-R energy parameter is not discussed further. Overall, the kinetic and isotherm analyses confirm the rapid uptake and high apparent Ba^2+^ capacity of HT-450, while the final immobilization mechanism is interpreted based on the structural and spectroscopic evidence presented below.

**Figure 5:**
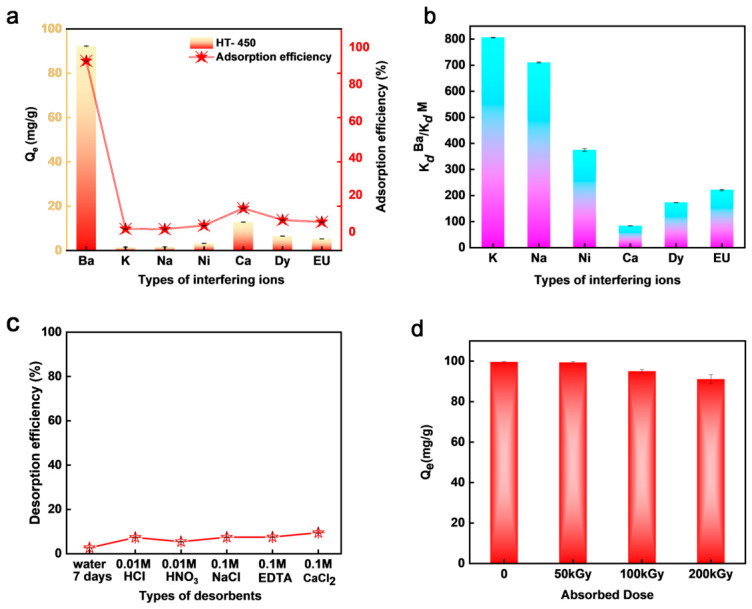
(**a**) Selectivity of HT-450 for Ba^2+^ adsorption in the presence of competing ions. (**b**) Separation factors (*K_d_*) of Ba^2+^ relative to coexisting ions on HT-450. (**c**) Desorption behavior of Ba^2+^ from HT-450 using different eluents. (**d**) Radiation tolerance of HT-450 under high-energy electron irradiation.

The selectivity of HT-450 toward Ba^2+^ was further evaluated in the presence of representative mono-, di-, and trivalent cations, including K^+^, Na^+^, Ca^2+^, Ni^2+^, Dy^3+^, and Eu^3+^. As shown in Figure 5a, HT-450 exhibited a markedly higher uptake capacity for Ba^2+^ than for all competing ions, including chemically similar Ca^2+^, indicating a pronounced preference for Ba^2+^ under multicomponent conditions [56]. This result is further supported by the high separation factors shown in Figure 5b. In these competitive ion experiments, each coexisting ion was initially set at 100 ppm without intentional addition of NaHCO_3_ or Na_2_CO_3_. Additional single-ion adsorption and ternary alkaline-earth competitive experiments further showed that HT-450 immobilized Ba^2+^ more favorably than Sr^2+^ and Ca^2+^ under comparable conditions (Tables S4 and S5), suggesting that the observed Ba^2+^ selectivity is more likely associated with the combined effects of ion-specific behavior, carbonate-mediated solid-phase formation, and stabilization by the reconstructed HT-450 matrix rather than carbonate precipitation tendency alone.

Although these model competitors do not fully represent the complexity of real radioactive wastewater matrices, the results nevertheless suggest promising selectivity under multicomponent conditions. Desorption experiments further demonstrate the high stability of immobilized Ba on HT-450. Extremely low release efficiencies are observed after soaking in deionized water for 7 days or after treatment with 0.01 M HCl, 0.01 M HNO_3_, 0.1 M NaCl, 0.1 M EDTA, or 0.1 M CaCl_2_ (Figure 5c), indicating that Ba remains strongly immobilized on HT-450 with extremely low remobilization under the tested conditions. Although these experiments do not by themselves establish very-long-term stability, they provide clear evidence for the strong short-term retention of immobilized Ba in the presence of both aqueous and chemical perturbations. Additional high-ionic-strength stability tests showed that Ba release remained below 5% in 0.5–1.0 M NaCl and NaNO_3_ solutions (Figure S5), further supporting the strong retention of immobilized Ba under concentrated chloride- and nitrate-rich conditions. The low Ba desorption indicates that conventional regeneration of spent HT-450 may be difficult; however, the primary purpose of this material is stable Ba^2+^ immobilization rather than reversible adsorption. For radioactive Ba^2+^-containing wastewater treatment, strong retention and low remobilization risk are highly desirable. Moreover, HT-450 can be prepared through a simple coprecipitation–calcination route using low-cost Mg-Al precursors, making it suitable for single-use immobilization applications. The spent Ba-loaded HT-450 should therefore be managed as a secondary solid waste and further stabilized/solidified according to radioactive waste management requirements.

The radiation tolerance of HT-450 was evaluated using an electron accelerator operated at 10 MeV, 20 kW, and 10 kGy s^−1^. The Ba^2+^ uptake performance was subsequently measured to determine whether the adsorption/immobilization function could be retained after irradiation. As shown in Figure 5d, HT-450 maintained nearly unchanged Ba^2+^ uptake capacities after irradiation up to 200 kGy, indicating excellent radiation tolerance and functional stability. To further probe the structural response to irradiation, XRD patterns of the irradiated samples were collected (Figure S6). All samples retained the characteristic diffraction features of the hydrotalcite-like phase, with no detectable impurity peaks or irradiation-induced phase transformation. Meanwhile, the gradual enhancement of the (003) reflection suggests improved structural ordering and partial reconstruction of the layered framework during irradiation. These results demonstrate that irradiation does not induce structural deterioration of HT-450, but instead preserves, and may even slightly improve, its structural integrity. Consistent with the adsorption results, HT-450 showed no obvious loss of Ba^2+^ removal performance after irradiation, confirming its suitability as a stable and radiation-tolerant adsorbent for Ba^2+^ removal from radioactive aqueous systems. Nevertheless, this electron-beam irradiation test should be regarded as an accelerated material-level screening experiment, and further studies under long-term low-dose-rate, mixed-radiation, aqueous radiolysis, and realistic wastewater chemistry conditions are still needed to fully evaluate the practical radiation stability of HT-450.

### 3.3. Adsorption Mechanism Analysis

Within the investigated pH range where homogeneous BaCO_3_ precipitation was negligible in the blank controls, the immobilization of Ba^2+^ on HT-450 is more appropriately interpreted as a predominantly reconstruction-coupled, interfacially mediated mineralization process rather than conventional reversible adsorption. Although kinetic and isotherm models are useful for describing the apparent uptake behavior (Figure 4d–g), multiple lines of structural and spectroscopic evidence indicate that Ba^2+^ is ultimately converted into a stable solid phase through carbonate-mediated BaCO_3_ formation closely associated with the HT-450 surface [57]. The carbonate species involved in this process are likely derived from dissolved inorganic carbon in the aqueous environment, including atmospheric CO_2_ absorbed during reconstruction, although this source assignment is inferred from the reaction conditions rather than directly quantified. To further evaluate the role of dissolved inorganic carbon, an N_2_-degassing experiment was conducted. The Ba^2+^ uptake capacity decreased from 416 mg g^−1^ under air-equilibrated conditions to 328 mg g^−1^ under N_2_-degassed conditions (Figure S7), suggesting that dissolved inorganic carbon contributes to Ba^2+^ immobilization. However, N_2_ degassing does not necessarily eliminate all dissolved inorganic carbon; therefore, the remaining uptake may be related to residual inorganic carbon/carbonate species and surface-associated interactions with the reconstructing HT-450 matrix. Because solid-phase XRD and FT-IR analyses under N_2_-degassed conditions were not performed, this result should be regarded as indirect evidence for the involvement of dissolved inorganic carbon rather than direct quantitative evidence for changes in BaCO_3_ formation. Structural and spectroscopic analyses were then conducted to clarify the immobilization pathway. Upon contact with aqueous solution, HT-450 rapidly undergoes partial structural reconstruction driven by the intrinsic memory effect of calcined LDHs. In the present system, calcination does not enhance Ba^2+^ uptake through classical cation exchange. Instead, it converts the parent LDH into a reactive mixed-oxide precursor with defect-rich surfaces, higher accessibility, and stronger reconstruction ability [58,59]. During rehydration, HT-450 generates fresh hydroxylated interfaces and reconstructed LDH-like domains, which facilitate rapid surface-associated contact of Ba^2+^ and subsequently promote carbonate-mediated interfacial mineralization into BaCO_3_. We therefore do not attribute the rapid Ba^2+^ uptake primarily to simple electrostatic attraction, but rather to a surface-associated interfacial process followed by mineralization and stabilization by the reconstructed LDH-like framework. The role of LDH-derived reconstruction was further supported by a non-LDH Mg–Al oxide control with the same Mg/Al ratio of 4:1, which was prepared and thermally treated at 450 °C for 8 h; this control showed no characteristic LDH basal reflections in XRD (Figure S8) and exhibited a much lower Ba^2+^ uptake capacity of approximately 54 mg g^−1^ than HT-450 (Table S6), indicating that the superior Ba^2+^ immobilization performance is closely associated with the LDH-derived reconstructable structure rather than Mg/Al composition or calcination temperature alone.

**Figure 6:**
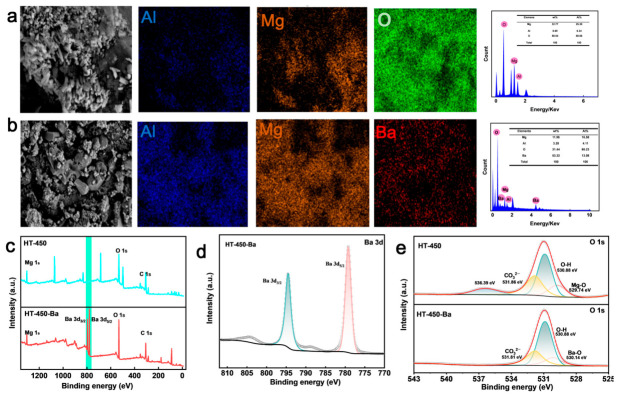
(**a**) SEM image of HT-450 and corresponding EDS elemental maps. (**b**) SEM image and EDS maps of HT-450 after Ba^2+^ adsorption. (**c**) XPS survey spectra of HT-450 before and after Ba^2+^ adsorption. High-resolution XPS spectra of (**d**) Ba 3d and (**e**) O 1s regions.

SEM-EDS analysis provides visual evidence for the immobilization process (Figure 6a,b). Pristine HT-450 exhibits a rough, aggregated morphology composed of irregularly stacked particles, with homogeneous distributions of Mg, Al, and O (Figure 6a). After Ba^2+^ treatment, the overall particle morphology remains largely unchanged, but a distinct Ba signal appears and is uniformly distributed across the particles (Figure 6b). No obvious Ba-rich agglomerates or detached precipitates are observed, suggesting that Ba is closely associated with the Mg-Al-O matrix and does not predominantly exist as large detached precipitate aggregates.

To distinguish interfacial BaCO_3_ mineralization from the loose attachment of externally generated BaCO_3_ particles, a control sample was prepared by first generating BaCO_3_ precipitates in a blank solution at pH 8 and then mixing them with HT-450. This pre-formed BaCO_3_/HT-450 mixture was compared with Ba-loaded HT-450 obtained from the normal adsorption experiment by ultrasonic stability testing. After ultrasonication for 15 min followed by acid digestion of the supernatant, only ~12% of Ba was released from Ba-loaded HT-450, whereas ~85% was released from the pre-formed BaCO_3_/HT-450 mixture (Figure S9). This large difference suggests that the Ba-containing phase formed during normal adsorption was more strongly retained by the reconstructed HT-450 matrix than externally generated BaCO_3_ particles simply mixed with HT-450.

XPS analysis further corroborates the successful immobilization of Ba^2+^ and provides information on its chemical environment (Figure 6c–e). The survey spectra clearly show the emergence of pronounced Ba 3d peaks after treatment (Figure 6c), confirming Ba uptake on the surface. High-resolution Ba 3d spectra display characteristic Ba 3d_5/2_ and Ba 3d_3/2_ peaks corresponding to Ba^2+^ (Figure 6d), indicating that no redox transformation occurs during the immobilization process. In the O 1s region, the main peak shifts toward higher binding energy after Ba^2+^ treatment (Figure 6e), consistent with the involvement of Ba-related oxygen species and stronger chemical association between immobilized Ba and the surface.

**Figure 7:**
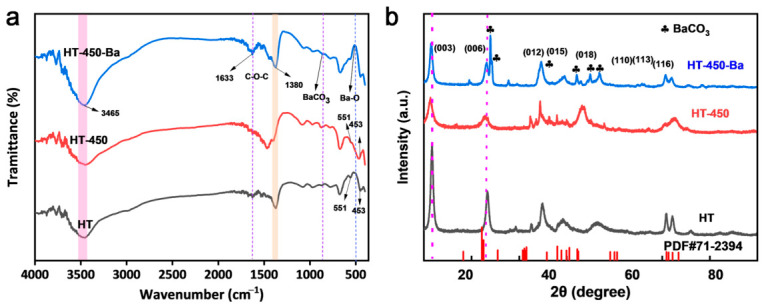
(**a**) FT-IR spectra and (**b**) XRD patterns of pristine HT, HT-450, and Ba^2+^-adsorbed HT-450.

FT-IR and XRD analyses provide key evidence for carbonate-mediated mineralization. FT-IR spectra reveal the disappearance of residual carbonate bands in HT-450 and the emergence of new vibrational features characteristic of BaCO_3_ after Ba^2+^ treatment (Figure 7a). XRD patterns of the Ba^2+^-loaded sample display distinct diffraction peaks corresponding to crystalline BaCO_3_ (Figure 7b), clearly confirming the formation of a BaCO_3_ phase during immobilization. Simultaneously, partial reconstruction of LDH-like domains occurs via the memory effect, as evidenced by regenerated layered structures in HRTEM (interplanar spacing of ~0.76 nm corresponding to the (003) plane, Figure S10). This reconstructed framework likely contributes to the stable retention of BaCO_3_ on the HT-450 surface. Ion exchange is unlikely to be the dominant pathway, as suggested by the limited Mg^2+^ leaching (Table S7) and the absence of clear evidence for substantial interlayer-spacing evolution in XRD (Figure 7b).

However, a minor contribution from surface-associated ion exchange cannot be completely excluded. Based on the combined evidence from blank control experiments (Figure S4), ultrasonic stability testing (Figure S9), SEM-EDS (Figure 6a,b), XPS (Figure 6c–e), FT-IR (Figure 7a), XRD (Figure 7b), HRTEM (Figure S10), and desorption experiments (Figure 5c), Ba^2+^ immobilization on HT-450 within the investigated pH range can be interpreted as a synergistic multi-step process: (i) rapid initial surface-associated interaction with the reconstructed or reconstructing mixed-oxide surface, (ii) carbonate-mediated interfacial mineralization leading to crystalline BaCO_3_ formation, and (iii) stabilization of the immobilized Ba phase by the reconstructed LDH-like framework. Homogeneous BaCO_3_ precipitation may become relevant under more alkaline conditions, such as pH 8, but it was negligible in the blank controls at pH 2–7. This mechanism accounts for the high apparent uptake capacity, fast kinetics, low remobilization under the tested conditions, and excellent radiation tolerance of the material.

## 4. Conclusions

In this work, Mg-Al layered double hydroxides were optimized by regulating the Mg/Al ratio and calcination temperature for efficient Ba^2+^ removal from radioactive wastewater. The optimized HT-450 exhibited rapid Ba^2+^ uptake, high removal capacity, strong selectivity, and good retention stability under the tested conditions. Structural, spectroscopic, control, and stability analyses suggest that Ba^2+^ immobilization is mainly associated with reconstruction-coupled, interfacially mediated BaCO_3_ mineralization rather than simple reversible adsorption or ion exchange. During hydration-driven reconstruction, the LDH-derived Mg-Al matrix likely provides favorable interfacial sites for Ba^2+^ association, carbonate-mediated solid-phase formation, and stabilization of the immobilized Ba-containing phase, thereby reducing remobilization risk.

Although HT-450 shows promising potential for stable Ba^2+^ immobilization, several aspects require further investigation before practical application, including the exact carbonate source involved in BaCO_3_ formation, long-term stability under realistic radioactive wastewater conditions, and the management or stabilization/solidification of the spent Ba-loaded adsorbent. Overall, the simple preparation route, low-cost Mg-Al precursors, rapid uptake, and strong Ba retention make HT-450 a promising material platform for radioactive Ba^2+^ immobilization and provide insight into LDH reconstruction-assisted carbonate mineralization for nuclear wastewater remediation.

## Data Availability

The original contributions presented in this study are included in this article. Further inquiries can be directed to the corresponding author.

## Supplementary Materials

The following supporting information can be downloaded at https://www.mdpi.com/article/10.3390/toxics14050432/s1, Figure S1. Comparison of Ba^2+^ uptake by different LDH systems before and after calcination; Figure S2. Effect of the Mg/Al molar ratio on the Ba^2+^ uptake capacity of HT-450; Figure S3. Effect of precursor interlayer anions on the Ba^2+^ uptake capacity of calcined LDHs; Figure S4. Ba^2+^ removal in blank solutions without HT-450 at different initial pH values; Figure S5. Ba release from Ba-loaded HT-450 under high-ionic-strength electrolyte conditions; Figure S6. XRD patterns of HT-450 after electron-beam irradiation at different doses; Figure S7. Effect of N_2_ degassing on Ba^2+^ uptake by HT-450; Figure S8. XRD pattern of the non-LDH Mg–Al oxide control; Figure S9. Ultrasonic stability comparison between Ba-loaded HT-450 and the pre-formed BaCO_3_/HT-450 mixture; Figure S10. TEM/HRTEM characterization of Ba-loaded HT-450; Table S1. Comparison of the adsorption performance of various materials toward Ba^2+^; Table S2. Kinetic fitting parameters for Ba^2+^ adsorption onto HT-450; Table S3. Isotherm fitting parameters for Ba^2+^ adsorption onto HT-450; Table S4. Single-ion uptake capacities of HT-450 toward Ba^2+^, Sr^2+^, and Ca^2+^; Table S5. Competitive uptake of Ba^2+^, Sr^2+^, and Ca^2+^ by HT-450 in a ternary system; Table S6. Comparison of Ba^2+^ uptake by HT-450, HT-800, and the non-LDH Mg–Al oxide control; Table S7. Amount of leached Mg^2+^ after contact of the getters with Ba^2+^ solution and H_2_O. References [6,14,17,60,61,62,63,64,65,66,67,68,69,70,71,72] are cited in the Supplementary Materials.
