# Supplementary material for: Highly Efficient Removal of Barium(II) from Nuclear Wastewater by Calcined Magnesium–Aluminum Layered Double Hydroxides
Source: Toxics. 2026 May 14;14(5):432. doi: 10.3390/toxics14050432 (PMC13211766; doi:10.3390/toxics14050432)
Supplement: Supplementary file 1 [file toxics-14-00432-s001.zip › toxics-4268134-supplementary.pdf]

Supporting Information

Highly Efficient Removal of Barium(II) from Nuclear Wastewater by Calcined Magnesium-Aluminum Layered Double Hydroxides

Jun Wang [1], Zhaoxu Sun [1], Ziyi Liu [1], Xinglei Li [1], Yi Zhou [1], Ningchao Zheng [1,*], Qiang Wu [1], Chen Xu [1], Lu Gao [2], Hiroshi Watabe [3], Yanliang Chen [4], Yuezhou Wei [1,5,*], and Xiangbiao Yin [1,*]

1 School of Nuclear Science and Technology, University of South China, 28 Changsheng West Road, Hengyang 421001, PR China.
2 School of Economics, Management and Law, University of South China, 28 Changsheng West Road, Hengyang 421001, PR China.
3 Research Center for Accelerator and Radioisotope Science (RARiS), Tohoku University, 6-3 Aoba, Aramaki, Aoba-ku, Sendai, Miyagi 980-8578, Japan.
4 Shanghai Institute of Measurement and Testing Technology Co., Ltd.
5 School of Nuclear Science and Engineering, Shanghai Jiao Tong University, 800 Dong Chuan Road, Shanghai 200240, PR China.

* Corresponding author: yzwei@usc.edu.cn(Y.W.),
yinxb@usc.edu.cn (X.Y.),

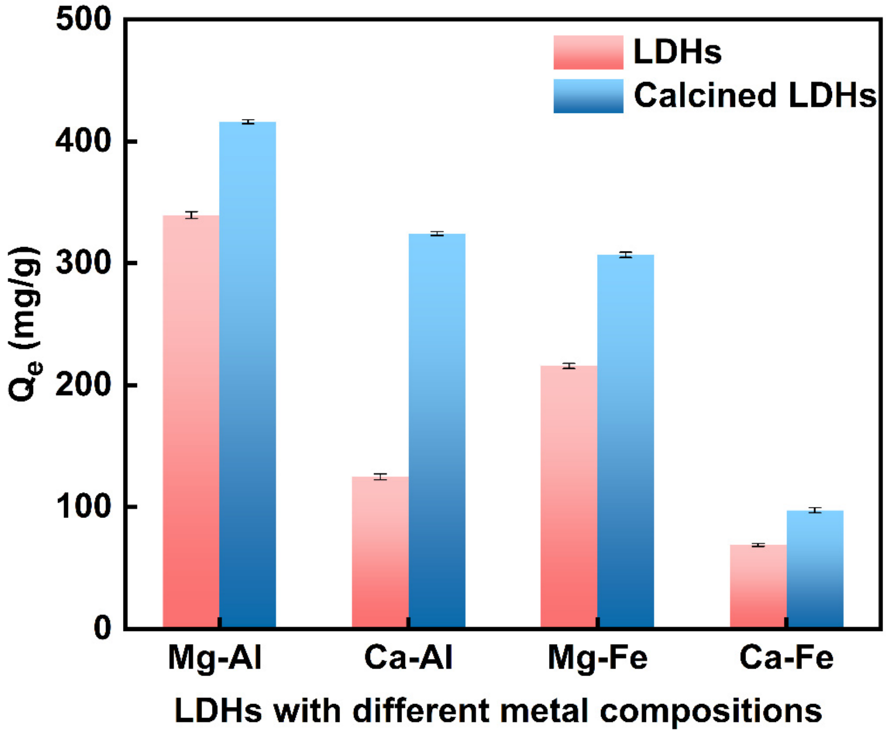

Fig. S1. Effect of Mg/Al molar ratio on the maximum removal capacity for Ba^{2+}.

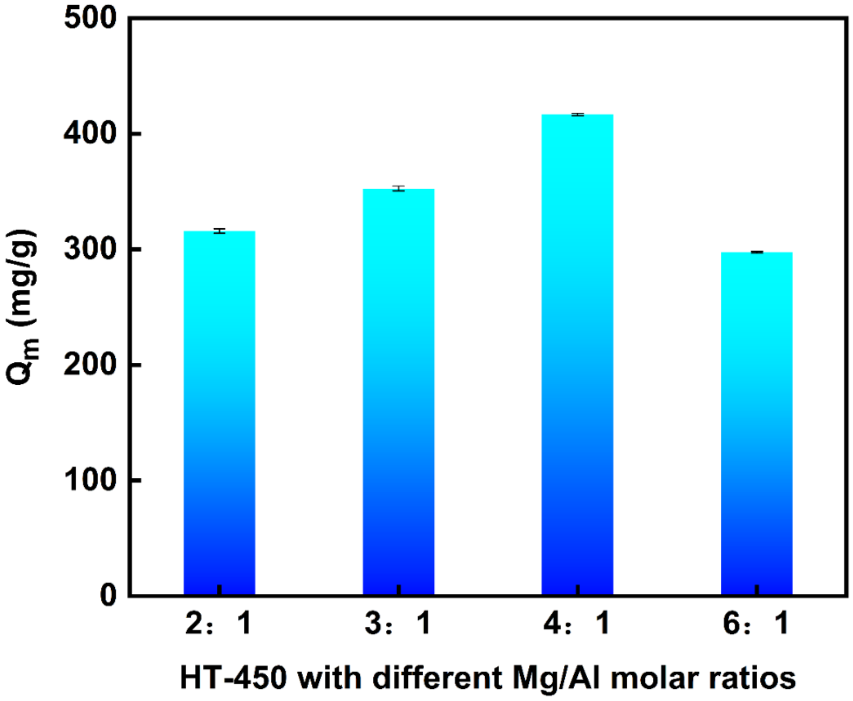

Fig. S2. Effect of Mg/Al molar ratio on the Ba^{2+} uptake capacity of HT-450.

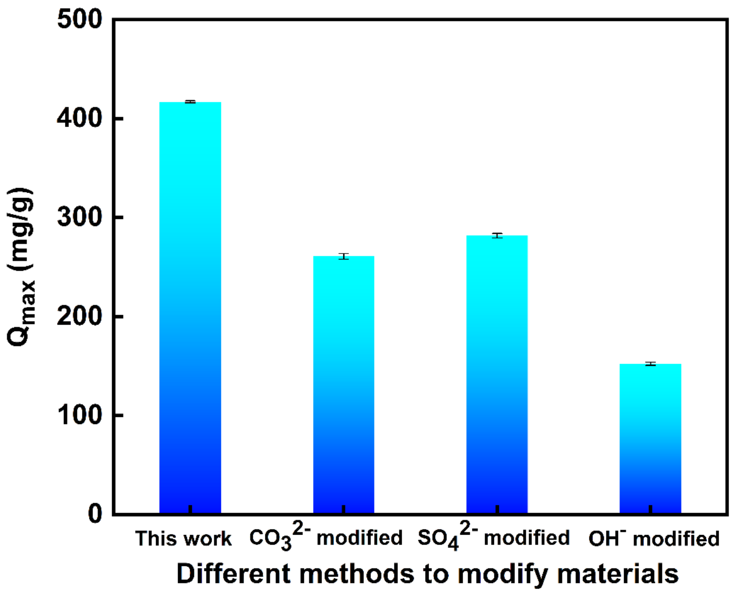

Fig. S3. Comparison of maximum Ba^{2+} removal capacities of HT-450 obtained in this work and LDHs modified by different anions.

The Ba^{2+} concentration remained essentially unchanged from pH 2 to 7, suggesting negligible homogeneous $BaCO_3$ precipitation within the pH range used for the mechanistic analysis. Noticeable Ba^{2+} loss was observed only at pH 8, indicating that homogeneous precipitation becomes more relevant under more alkaline conditions.

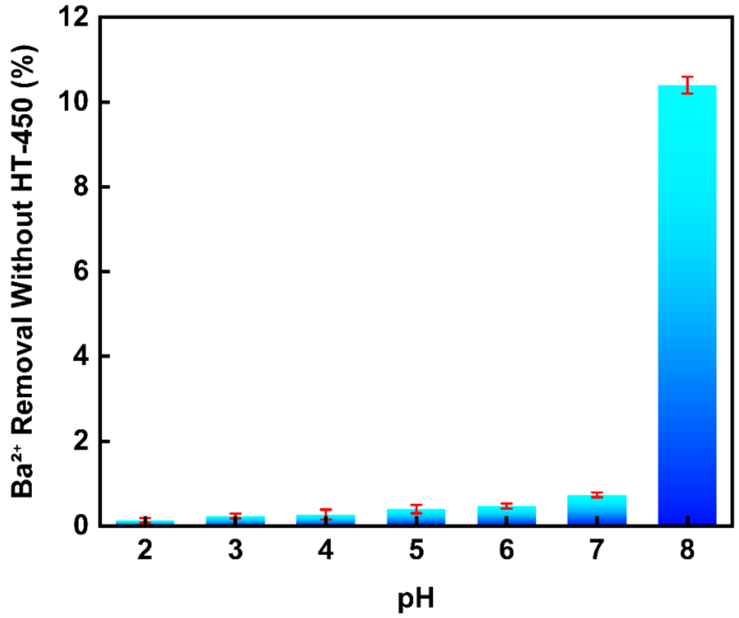

Fig. S4. Ba^{2+} removal in blank solutions without HT-450 at different initial pH values under otherwise identical experimental conditions.

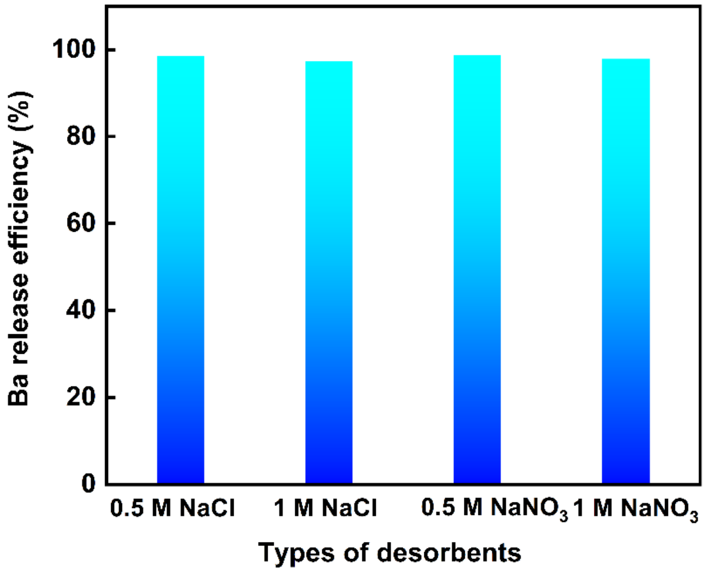

Fig. S5. Ba release from Ba-loaded HT-450 under high-ionic-strength electrolyte conditions.

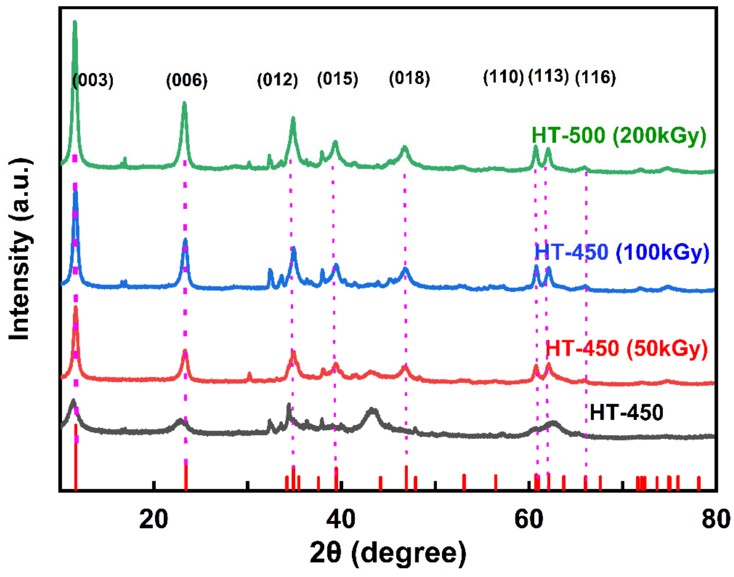

Fig. S6. XRD patterns of HT-450 after electron-beam irradiation at different doses.

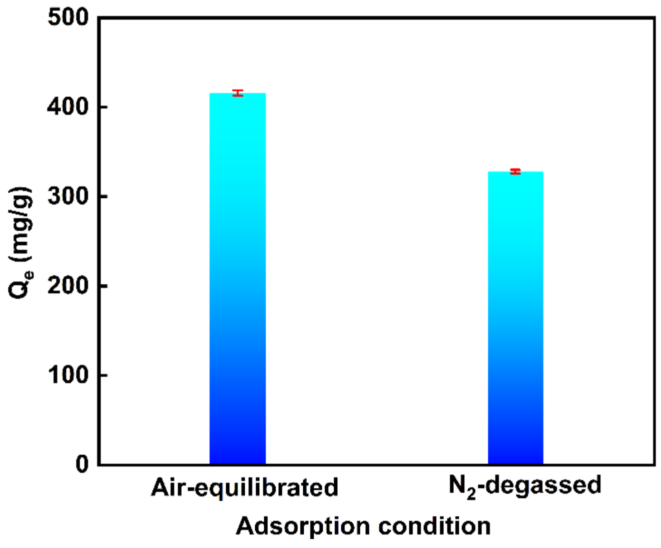

Fig. S7. Effect of N_2 degassing on Ba^{2+} uptake by HT-450.

N_2-degassing experiment: To evaluate the influence of dissolved inorganic carbon, the Ba^{2+} solution was purged with N_2 before adsorption. HT-450 was then added under otherwise identical adsorption conditions, and the residual Ba^{2+} concentration was measured by ICP-OES after reaction.

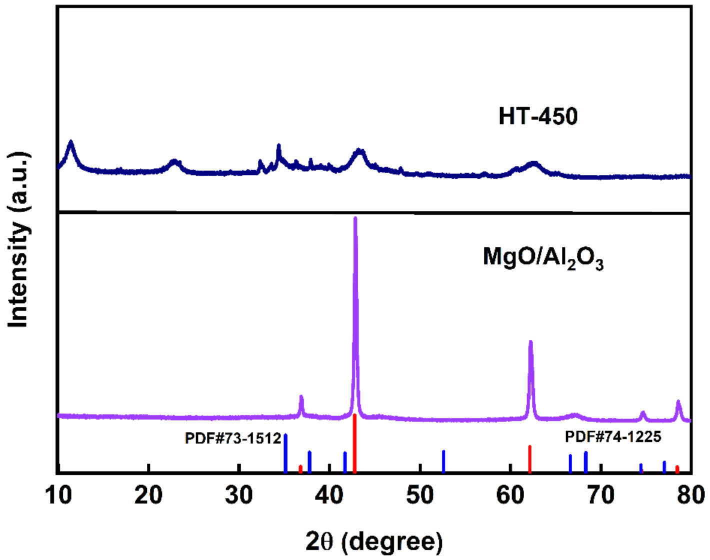

Fig. S8. XRD pattern of the non-LDH Mg–Al oxide control with the same Mg/Al molar ratio of 4:1 after thermal treatment at 450 °C for 8 h. No characteristic LDH basal reflections were observed, confirming the absence of an LDH layered structure in this control sample.

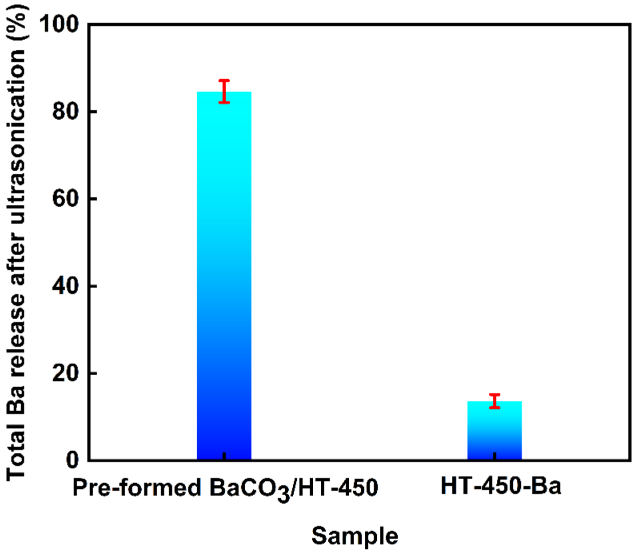

Fig. S9. Ultrasonic stability comparison between Ba-loaded HT-450 and the pre-formed $BaCO_3$/HT-450 mixture.

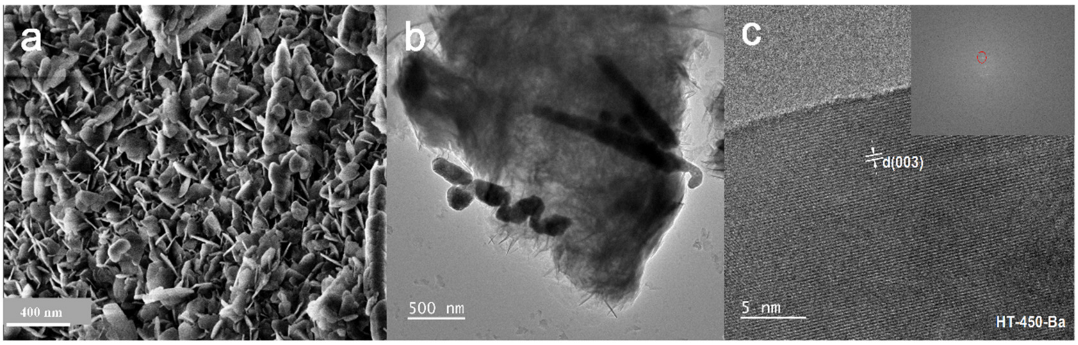

Fig. S10. (a) SEM image of HT-450 after Ba^{2+} adsorption, showing the aggregated and roughened surface morphology after Ba^{2+} immobilization. (b) TEM image of HT-450-Ba, revealing the formation of irregularly aggregated oxide domains after adsorption. (c) HRTEM image of HT-450-Ba, displaying clear lattice fringes with an interplanar spacing of d = 0.76 nm, corresponding to the (003) plane of reconstructed LDH domains.

Table S1. Comparison of adsorption performance for Ba^{2+} over various materials.

Adsorbents	Q_e(mg g^{-1})	Time (h)	pH	References
Aloe Vera biosorbent	107.5	1	5.0	[60]
Ca-clinoptilolite	15.3	168	-	[17]
Ca-montmorillonite	15.3	48	-	[17]
Calcined Hydrotalcite	360.0	-	-	[61]
Dolomite	4.0	2	5.5	[6]
Expanded perlite	2.5	1.5	6.0	[62]
Fungus-titanate bio-nanocomposites	120	1	11.4	[14]
MIL-101-Cr-SO_3H	70.5	-	-	[63]

MOF-808-SO_4	131.1	0.1	5.8	[63]
$Na_2Ti_3O_7$	159.6	-	-	[64]
Natural allophane	10.6	0.2	8.5	[65]
Natural clinoptilolite	41.1	72	-	[66]
Na-4-mica	78.8	-	-	[64]
Na-clinoptilolite	109.6	73	-	[66]
Nano-polymer SAB	57.7	8	8.0	[67]
Nano-polymer SASB	210.4	6	8.0	[67]
Synthetic allophane-1	38.6	0.2	8.5	[65]
Synthetic allophane-2	17.2	0.2	8.5	[65]
$Ti_3C_2T_X$	12.0	24	7.0-10.0	[68]
Zeolite-rich tuff	230.2	0.5	7.7	[69]
Zeolite Z70-4	17.0	0.5	4.0-5.0	[70]
Zeolite Z90-4	119.0	0.5	4.0-5.0	[70]
Zeolite Z90-15	117.7	0.5	4.0-5.0	[70]
Zero-valent iron	22.6	-	-	[71]
Zr-BDC-NH_2-SO_4	181.8	-	-	[72]
This work	416.0	0.3	3-7	

Table S2. Kinetic fitting parameters of HT-450 for Ba^{2+} adsorption.

Kinetic model	Parameter	
Pseudo-first-order	Q_{e1} (mg g^{-1})	444.23
	K_1 (min^{-1})	0.22
	R^2	0.909
Pseudo-second-order	Q_{e2} (mg g^{-1})	426.8
	K_2 (g mg^{-1} min^{-1})	0.001
	R^2	0.996
Intraparticle diffusion	K_{p1} (mg g^{-1} $min^{-1/2}$)	2.01
	K_{p2} (mg g^{-1} $min^{-1/2}$)	31.4
	K_{p3} (mg g^{-1} $min^{-1/2}$)	0.35

C_1 (mg g^{-1})	146.09
C_2 (mg g^{-1})	236.59
C_3 (mg g^{-1})	411.99

Table S3. Isotherm fitting parameters of HT-450 for Ba^{2+} adsorption.

Isotherm model	Parameter	Ba^{2+}
Langmuir	Q_m (mg g^{-1})	421.13
	K_L (L mg^{-1})	0.003
	R^2	0.926
Freundlich	n	3.59
	K_F (mg/g)	289.32
	R^2	0.838
Dubinin-Radushkevich	Q_{e3} (mg/g)	289.78
	E	14.17
	R^2	0.77

Table S4. Single-ion uptake capacities of HT-450 toward Ba^{2+}, Sr^{2+}, and Ca^{2+} at an initial concentration of 1000 ppm.

Ion	Initial concentration (ppm)	Uptake capacity (mg g^{-1})
Ba^{2+}	1000	416
Sr^{2+}	1000	238
Ca^{2+}	1000	175

Table S5. Competitive uptake of Ba^{2+}, Sr^{2+}, and Ca^{2+} by HT-450 in a ternary system.

Ion	Initial concentration	Removed concentration	Removal efficiency
Ba^{2+}	1 mmol L^{-1}	126 mg L^{-1}	91.8%
Sr^{2+}	1 mmol L^{-1}	46 mg L^{-1}	52.5%
Ca^{2+}	1 mmol L^{-1}	15 mg L^{-1}	37.4%

Table S6. Comparison of Ba^{2+} uptake by HT-450, HT-800, and the non-LDH Mg–Al oxide control.

Sample	Key structural feature	Ba^{2+} uptake capacity (mg g^{-1})
HT-450	LDH-derived reconstructable oxide	416
HT-800	Over-calcined oxide with weakened/absent reconstruction ability	125
Non-LDH Mg–Al oxide	Same Mg/Al ratio; no LDH basal reflections	54

Table S7. Amount of leached Mg^{2+} after mixing the getters with excessive amount of target metal ions (25 ml, 2000 mg/L of Ba^{2+} solutions and H_2O) using HT and HT-450.

Getters	Mg^{2+} Leaching Amount (mg g-1)	
	Ba^{2+}	Blank(H_2O)
HT	6.54	0.76
HT-450	0.07	0.01

SM References:

6 Ghaemi, A.; Torab-Mostaedi, M.; Ghannadi-Maragheh, M. Characterizations of strontium(II) and barium(II) adsorption from aqueous solutions using dolomite powder. *J. Hazard. Mater.* **2011**, *190*, 916–921. https://doi.org/10.1016/j.jhazmat.2011.04.006

14 Xu, M.Z.; Wei, G.D.; Liu, N.; Zhou, L.; Fu, C.W.; Chubik, M.; Gromov, A.; Han, W. Novel fungus-titanate bio-nanocomposites as high performance adsorbents for the efficient removal of radioactive ions from wastewater. *Nanoscale* **2014**, *6*, 722–725. https://doi.org/10.1039/c3nr03467d

17 Chávez, M.L.; de Pablo, L.; García, T.A. Adsorption of Ba^{2+} by Ca-exchange clinoptilolite tuff and montmorillonite clay. *J. Hazard. Mater.* **2010**, *175*, 216–223. https://doi.org/10.1016/j.jhazmat.2009.09.151.

60. Kapashi, E.; Kapnisti, M.; Dafnomili, A.; Noli, F. AloeVera as an effective biosorbent for the removal of thorium and barium from aqueous solutions. *J. Radioanal. Nucl. Chem.* **2019**, *321*, 217–226.

61. Bo, A.; Sarina, S.; Liu, H.W.; Zheng, Z.F.; Xiao, Q.; Gu, Y.T.; Ayoko, G.A.; Zhu, H.Y. Efficient removal of cationic and anionic radioactive pollutants from water using hydrotalcite-based getters. *ACS Appl. Mater. Interfaces* **2016**, *8*, 16503–16510.

62. Torab-Mostaedi, M.; Ghaemi, A.; Ghassabzadeh, H.; Ghannadi-Maragheh, M. Removal of strontium and barium from aqueous solutions by adsorption onto expanded perlite. *Can. J. Chem. Eng.* **2011**, *89*, 1247–1254.

63. Peng, Y.G.; Huang, H.L.; Liu, D.H.; Zhong, C.L. Radioactive barium ion trap based on metal-organic framework for efficient and irreversible removal of barium from nuclear wastewater. *ACS Appl. Mater. Interfaces* **2016**, *8*, 8527–8535.
64. Yang, D.J.; Zheng, Z.F.; Zhu, H.Y.; Liu, H.W.; Gao, X.P. Titanate nanofibers as intelligent absorbents for the removal of radioactive ions from water. *Adv. Mater.***2008**, *20*, 2777-2781.
65. Baldermann, A.; Griebacher, A.C.; Baldermann, C.; Purgstaller, B.; Dietzel, M. Removal of barium, cobalt, strontium and zinc from solution by natural and synthetic allophane adsorbents. *Geosciences* **2018**, *8*, 309.
66. Faghihian, H.; Marageh, M.G.; Kazemian, H. The use of clinoptilolite and its sodium form for removal of radioactive cesium and strontium from nuclear wastewater and Pb^{2+}, Ni^{2+}, Cd^{2+}, Ba^{2+} from municipal wastewater. *Appl. Radiat. Isot.* **1999**, *50*, 655–660.
67. Younis, S.A.; Ghobashy, M.M.; Bassioni, G.; Gupta, A.K. Tailored functionalized polymer nanoparticles using gamma radiation for selected adsorption of barium and strontium in oilfield wastewater. *Arab. J. Chem.* **2020**, *13*, 3762–3774.
68. Mu, W.J.; Du, S.Z.; Yu, Q.H.; Li, X.L.; Wei, H.Y.; Yang, Y.C. Improving barium ion adsorption on two-dimensional titanium carbide by surface modification. *Dalton Trans.* **2018**, *47*, 8375–8381.
69. De Gennaro, B.; Pepe, F.A.P.; Caputo, D. Natural zeolites for heavy metals removal from aqueous solutions: Modeling of the fixed bed Ba^{2+}/Na^{+} ion-exchange process using a mixed phillipsite/chabazite-rich tuff. *Chem. Eng. J.* **2013**, *219*, 37–42.
70. Noli, F.; Kapnisti, M.; Buema, G.; Harja, M. Retention of barium and europium radionuclides from aqueous solutions on ash-based sorbents by application of radiochemical techniques. *Appl. Radiat. Isot.* **2016**, *116*, 102–109.
71. Celebi, O.; Uzüm, C.; Shahwan, T.; Erten, H.N. A radiotracer study of the adsorption behavior of aqueous Ba^{2+} ions on nanoparticles of zero-valent iron. *J. Hazard. Mater.* **2007**, *148*, 761–767.
72. Kang, C.; Peng, Y.; Tang, Y.; Huang, H.; Zhong, C. Sulfate-rich metal–organic framework for high efficiency and selective removal of barium from nuclear wastewater. *Ind. Eng. Chem. Res.* **2017**, *56*, 13866–13873.
